# Supplementary material for: Creating Understandable and Actionable COVID-19 Health Messaging for Refugee, Immigrant, and Migrant Communities
Source: Healthcare (Basel). 2023 Apr 12;11(8):1098. doi: 10.3390/healthcare11081098 (PMC10137961; doi:10.3390/healthcare11081098)
Supplement: Supplementary file 1 [file healthcare-11-01098-s001.zip › healthcare-2301655-supplementary.pdf]

When can my child get a booster?

All children, ages 5-17

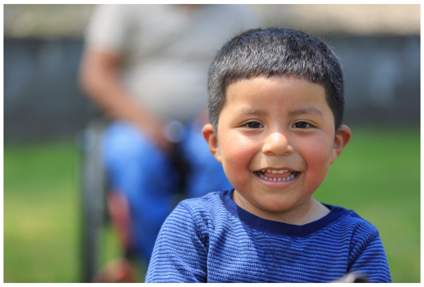

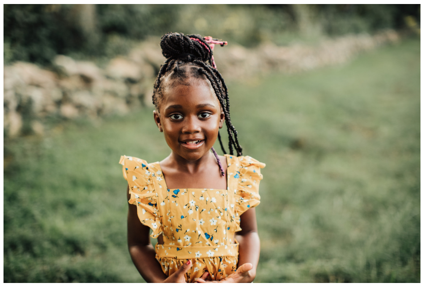

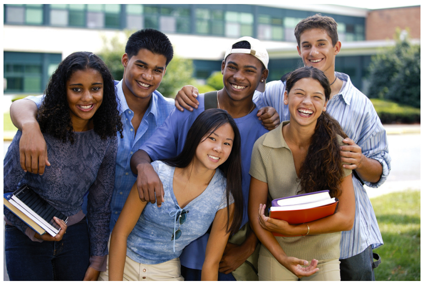

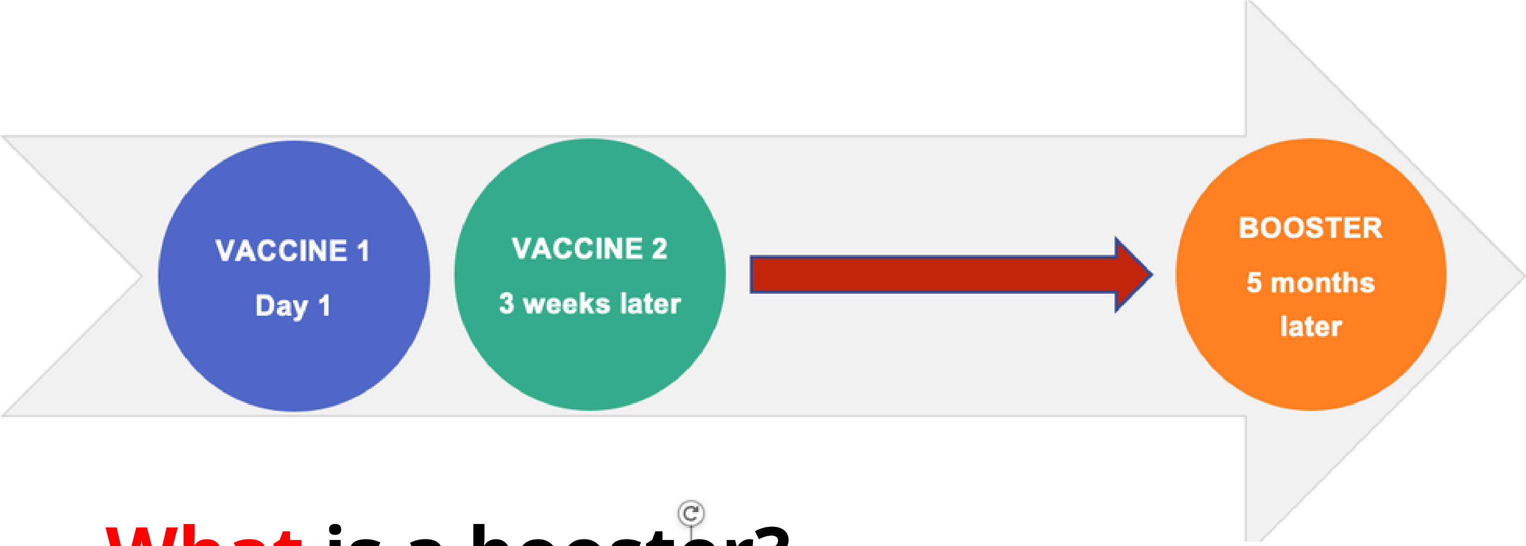

What is a booster?

A booster is a third shot of the COVID-19 vaccine. It helps build up more protection against COVID-19.

Why does my child need a booster?

Protection against COVID-19 from the first two shots can shrink over time. The booster increases your child's safety against getting sick with COVID-19.

[Example of requested flyer to help communities understand booster vaccination for children]

Georgia State University | PRC PREVENTION RESEARCH CENTERS

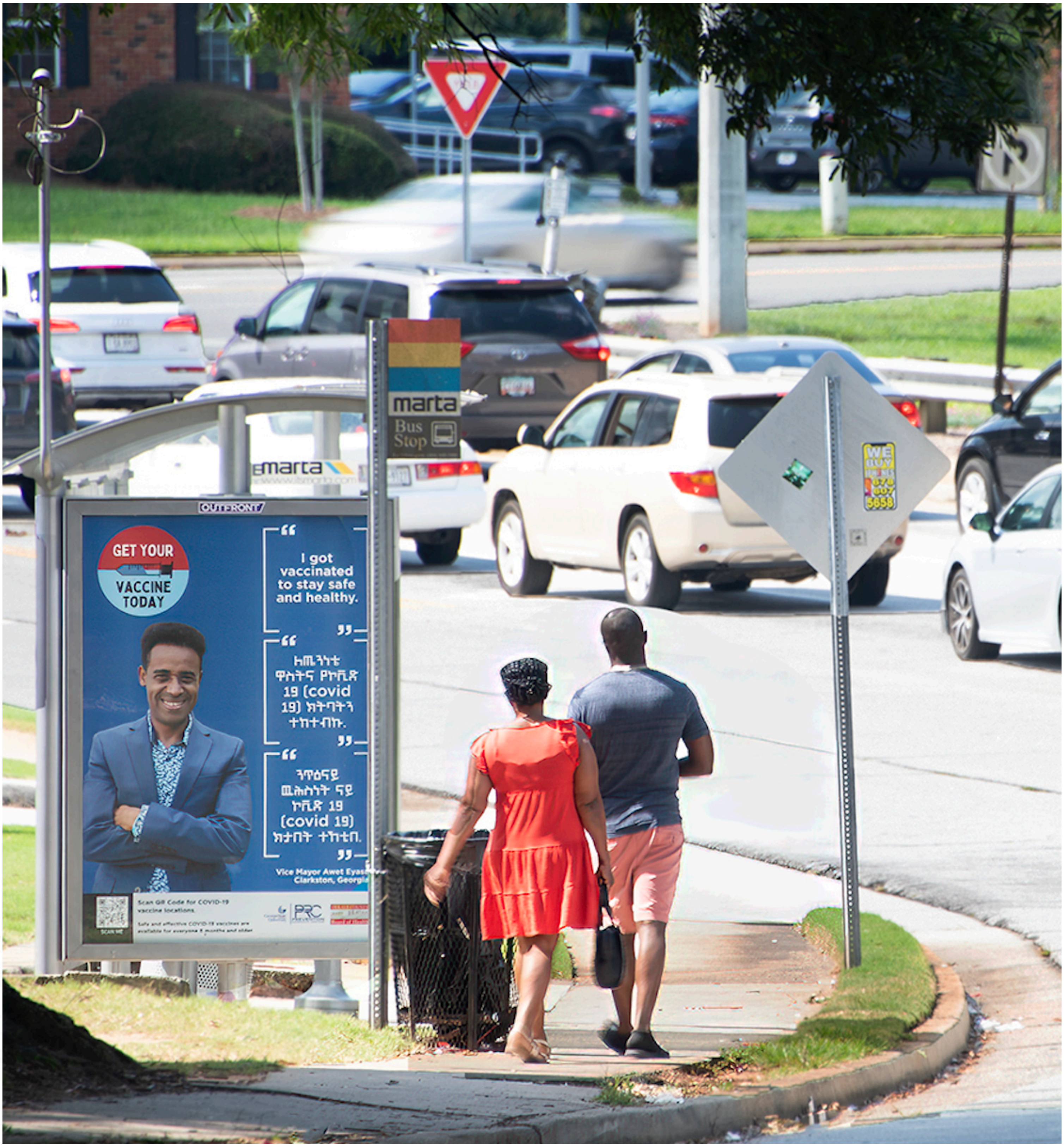

Example of Bus Stop Campaign Ad targeted to area frequented by Ethiopian Community (English, Amharic, Tigrinya)
